# Breaking Bones, Breaking Barriers: ChatGPT, DeepSeek, and Gemini in Hand Fracture Management

**DOI:** 10.3390/jcm14061983

**Published:** 2025-03-14

**Authors:** Gianluca Marcaccini, Ishith Seth, Yi Xie, Pietro Susini, Mirco Pozzi, Roberto Cuomo, Warren M. Rozen

**Affiliations:** 1 Plastic Surgery Unit, Department of Medicine, Surgery and Neuroscience, University of Siena, 53100 Siena, Italy; gianlu32@gmail.com (G.M.); susinipietro@gmail.com (P.S.); mircovirgilio.pozzi@gmail.com (M.P.); roberto.cuomo@unisi.it (R.C.); 2 Department of Plastic and Reconstructive Surgery, Peninsula Health, Frankston, VIC 3199, Australia; philip.y.xie@gmail.com (Y.X.); warrenrozen@hotmail.com (W.M.R.)

**Keywords:** hand fractures, artificial intelligence, large language models, ChatGPT-4o, DeepSeek-V3, Gemini 1.5, surgical decision-making

## Abstract

**Background**: Hand fracture management requires precise diagnostic accuracy and complex decision-making. Advances in artificial intelligence (AI) suggest that large language models (LLMs) may assist or even rival traditional clinical approaches. This study evaluates the effectiveness of ChatGPT-4o, DeepSeek-V3, and Gemini 1.5 in diagnosing and recommending treatment strategies for hand fractures compared to experienced surgeons. **Methods**: A retrospective analysis of 58 anonymized hand fracture cases was conducted. Clinical details, including fracture site, displacement, and soft-tissue involvement, were provided to the AI models, which generated management plans. Their recommendations were compared to actual surgeon decisions, assessing accuracy, precision, recall, and F1 score. **Results**: ChatGPT-4o demonstrated the highest accuracy (98.28%) and recall (91.74%), effectively identifying most correct interventions but occasionally proposing extraneous options (precision 58.48%). DeepSeek-V3 showed moderate accuracy (63.79%), with balanced precision (61.17%) and recall (57.89%), sometimes omitting correct treatments. Gemini 1.5 performed poorly (accuracy 18.97%), with low precision and recall, indicating substantial limitations in clinical decision support. **Conclusions**: AI models can enhance clinical workflows, particularly in radiographic interpretation and triage, but their limitations highlight the irreplaceable role of human expertise in complex hand trauma management. ChatGPT-4o demonstrated promising accuracy but requires refinement. Ethical concerns regarding AI-driven medical decisions, including bias and transparency, must be addressed before widespread clinical implementation.

## 1. Introduction

Hand trauma represents a critical and highly specialized domain within reconstructive and orthopedic surgery, often demanding precise diagnostic acumen and advanced surgical skills. The clinical management of hand fractures involves restoring skeletal integrity and preserving critical structures such as tendons, nerves, and vasculature, which are essential for functional recovery. Even seemingly straightforward fractures can harbor subtleties that influence surgical decision-making and rehabilitation protocols. In this context, achieving optimal outcomes requires a comprehensive understanding of hand anatomy, fracture mechanics, and the psychosocial impact on patients who depend heavily on manual dexterity for daily activities and employment.

Recent advancements in artificial intelligence (AI) suggest that machine learning algorithms may hold significant potential to support and, in some scenarios, potentially rival traditional approaches to managing hand fractures [1]. Innovations in deep learning have facilitated the automated detection and classification of skeletal injuries in medical imaging, sometimes with diagnostic accuracies comparable to those of experienced clinicians. In the broader fields of plastic and reconstructive surgery, systematic reviews underscore AI’s ability to achieve diagnostic performance on par with human clinicians, highlighting both the rapid computational capabilities of neural networks and the expanding volume of medical imaging data available for algorithmic training [2,3]. However, the question remains whether these emerging technologies truly match the nuanced decision-making, tactile feedback, and operative judgment of an experienced hand surgeon, particularly when confronted with complex scenarios such as multi-fragment fractures, tendon or nerve compromise, and extensive soft-tissue damage.

To explore this question, this retrospective analysis critically compares AI-driven fracture diagnosis and treatment planning from three distinct AI systems, ChatGPT-4o, DeepSeek-V3, and Gemini 1.5, with the real-world expertise of hand surgeons who have managed a range of traumatic injuries [4,5]. On the one hand, meta-analyses and prospective studies in orthopedic and reconstructive specialties provide promising evidence of AI’s capacity to match or exceed human-level accuracy in radiographic interpretation [6,7,8,9]. These tools also offer potential benefits in workflow optimization, such as rapidly triaging high-priority cases and leveraging predictive analytics to forecast complications, for instance, delayed union or infection [10]. On the other hand, treating hand trauma involves a series of intricate clinical judgments that extend beyond the scope of most current AI models.

Crucially, while AI-based approaches can streamline workflow and refine predictive analytics, their integration raises key concerns about algorithmic bias, data quality, and ethical frameworks for patient autonomy and informed consent [11,12,13,14,15,16,17]. In this comparative perspective, we highlight AI’s potential and limitations by juxtaposing specific clinical scenarios ranging from simple metacarpal fractures to complex hand injuries involving microvascular repair across three AI platforms: ChatGPT-4o, DeepSeek-V3, and Gemini 1.5 [18].

## 2. Materials and Methods

In this retrospective analysis, fifty-eight anonymized cases of hand fractures were identified from our institutional database, encompassing a variety of mechanisms (e.g., punch injuries, sports-related trauma, crush accidents) and diverse patient demographics. Pertinent clinical details characterized each case, including the precise fracture site (metacarpals or phalanges), associated soft-tissue damage (such as nailbed lacerations), the presence or absence of comminution or displacement, and any relevant comorbidities. Ethical approval was not required, as all patients had already undergone clinical management prior to this study. As a result, there was no potential for harm or risk to patients, and all data were fully de-identified to maintain confidentiality. This study was conducted according to the Declaration of Helsinki, with institutional ethical approval obtained from Victorian Ethics Review Management (LNR/97071/PH-2023).

Using these de-identified case profiles, we prompted three AI systems, ChatGPT-4o, DeepSeek-V3, and Gemini 1.5, to propose comprehensive management strategies. These strategies included preoperative evaluation, surgical or conservative treatment options, selection of fixation techniques (such as K-wire, plating, or dynamic external fixation), and any recommended ancillary procedures (such as tendon or nailbed repairs). The AI-generated plans were then compared directly to the definitive management documented by experienced hand surgeons, who employed a range of established operative methods, including the GAMP technique, Suzuki external fixation, and open reduction with internal fixation, as well as adjunct procedures for open fractures or soft-tissue repair. A comparative table was created to compile the fracture details, each AI model’s proposed approach, and the interventions performed for each patient. Discrepancies between AI recommendations and surgeon decisions regarding the choice of hardware, the necessity and timing of debridement, and postoperative immobilization were meticulously recorded. Ethical safeguards were implemented, ensuring that all patient identifiers were removed before review and that confidentiality and compliance with ethical research standards were maintained. The primary aim of this study was to evaluate the extent to which AI-generated treatment pathways align with routine clinical practice in the acute management of hand fractures, thereby assessing the potential of these AI systems to support and enhance surgical decision-making.

## 3. Results

The analysis of the three AI systems, ChatGPT, DeepSeek, and Gemini, reveals distinct performance profiles based on their accuracy, precision, recall, and F1 score metrics. ChatGPT demonstrates the highest accuracy at 98.28%, coupled with a robust recall of 91.74%, indicating its exceptional ability to capture the majority of the correct codes across cases. Its precision of 58.48% suggests that although it generally identifies correct codes, the multiple options it proposes sometimes include extraneous elements. In contrast, DeepSeek achieves a moderate accuracy of 63.79% with a precision of 61.17%, reflecting a high likelihood that its proposed codes are correct; however, its slightly lower recall of 57.89% indicates that it might overlook some correct codes. Gemini, on the other hand, performs notably lower across all metrics, with an accuracy of 18.97%, precision of 12.60%, recall of 14.68%, and an F1 score of 13.55%, indicating significant challenges in accurately predicting the correct codes. These results suggest that while ChatGPT excels in comprehensive code identification, its tendency to offer multiple options can affect precision, whereas DeepSeek provides more focused predictions at the cost of missing some correct codes and Gemini requires substantial improvements to enhance its predictive capabilities (Table 1, Table 2 and Table 3).

### Statistical Analysis

Performance metrics, including accuracy, precision, recall, and F1 score, were calculated according to standard definitions in the literature. These calculations were implemented using custom Python scripts (Python version 3.8 or later) that leveraged the NumPy and scikit-learn libraries for data processing and metric computation. To ensure transparency and reproducibility, the formulas were cross-validated using both automated code verification and manual review. In addition, ChatGPT (version 4o) was employed as an auxiliary tool to generate and verify portions of the computational code, further supporting the robustness of our methodology. This hybrid approach, integrating automated processes with expert oversight, confirmed the accuracy of the obtained values and provided thorough documentation of the entire analytical workflow. All results were subsequently subjected to quality control checks to ensure their consistency and reliability.

## 4. Discussion

The comparative analysis of fifty-eight surgical cases evaluating three artificial intelligence systems, ChatGPT, DeepSeek, and Gemini, reveals significant differences in their performance metrics, namely accuracy, precision, recall, and F1 score. These findings hold substantial implications for integrating AI-driven decision support tools in clinical settings, particularly in managing hand fractures. ChatGPT demonstrated exceptional accuracy at 98.28% and a notably strong recall of 91.74%, indicating its proficiency in identifying the majority of correct codes. This aligns with broader trends in plastic and reconstructive surgery literature, where AI systems have shown promise in matching or even exceeding human diagnostic and therapeutic accuracy [18,19]. ChatGPT’s ability to propose multiple codes increases the likelihood of including the correct intervention, thereby enhancing recall. However, this tendency also results in a precision of 58.48%, reflecting the inclusion of extra or incorrect options that can lower specificity. This balance underscores ChatGPT’s strength in comprehensive case coverage while highlighting a need for refinement to reduce erroneous predictions further.

DeepSeek exhibited a moderate accuracy of 63.79% and a precision of 61.17%, suggesting that its proposed codes are frequently correct. However, its recall stood at 57.89%, indicating that it may have missed some correct interventions, an aspect that can be critical in complex cases requiring exhaustive management strategies. This performance aligns with findings by Kuo et al., who noted that AI systems can achieve diagnostic and therapeutic accuracy comparable to human clinicians, albeit with variability in precision and comprehensiveness [1]. DeepSeek’s balanced but not robust precision and recall suggest it can serve as a reliable adjunct in clinical decision-making without overwhelming clinicians with excessive options. Nevertheless, its lower recall indicates that certain correct codes might be overlooked, necessitating complementary strategies to ensure broad coverage in clinical practice.

Gemini’s performance was notably lower across all metrics, with an accuracy of 18.97%, a precision of 12.60%, and a recall of 14.68%. This underscores significant challenges in its implementation, suggesting that Gemini struggles to accurately predict correct codes, rendering it less reliable for clinical decision support. Such underperformance mirrors concerns raised by Nogueira et al., who emphasized the risk of algorithmic oversimplification in complex clinical scenarios [2]. The limited effectiveness of Gemini underscores the critical need for advanced training algorithms, larger and more diverse datasets, and potentially different architectures to enhance its predictive capabilities. Gemini may not be suitable for clinical use without substantial improvements, particularly in intricate cases that demand nuanced decision-making.

From a diagnostic standpoint, the results reinforce the literature indicating that AI-based systems like ChatGPT and DeepSeek are capable of effective fracture recognition and risk stratification. Reviews in facial and reconstructive surgery have similarly documented AI’s strengths in rapid imaging analysis and predictive modeling (Espinosa Reyes et al. [3], Souza et al. [5]). In the context of hand trauma, these capabilities may translate into improved triage, expedited operative decision-making, and more targeted postoperative surveillance. However, the application of AI to complex upper-limb fractures remains challenging. Cases involving fragment comminution, concomitant tendon injuries, or crush components demand operative flexibility that current AI systems cannot yet fully replicate. Consistent with Maita et al.’s findings in reconstructive procedures [6], this underscores the necessity for tailored algorithms that accommodate a broad range of clinical variables rather than adopting a one-size-fits-all approach.

Ethical considerations remain paramount in the integration of AI into clinical decision-making. Echoing Pressman et al., any expansion of AI must address potential algorithmic biases, data privacy concerns, and the preservation of patient autonomy [17,20,21,22]. Our study, involving a limited sample size and a single AI platform for each system, highlights the necessity for further research with larger datasets and more diverse clinical scenarios to validate these preliminary findings. There is also a pressing need for standardized guidelines, as advocated by Wah and colleagues, who caution that disparities in AI access could exacerbate existing gaps in patient care [18]. Furthermore, the potential for racial or demographic bias, as raised by Haider et al. [21,22,23,24], emphasizes the importance of representative training data to ensure equitable AI performance across different patient populations.

This study has several limitations that must be acknowledged. Firstly, the analysis was based on a relatively small sample size of fifty-eight cases, which may limit the generalizability of the findings. Additionally, the reliance on case reports and the encoding of multiple proposed codes using a “+” delimiter present methodological challenges that could influence the accuracy and precision metrics observed. The retrospective design of the study and the inherent variability in case complexity also add layers of complexity that may affect the performance evaluation of the AI systems. Furthermore, the study utilized one instance of each AI platform, which may not capture the full breadth of capabilities and variations that different implementations of the same systems could exhibit.

Future research should address these limitations to provide a more comprehensive understanding of AI performance in clinical decision support. Larger and more diverse datasets are essential for enhancing the robustness and generalizability of the findings, and prospective studies or randomized controlled trials could offer more definitive evidence of AI efficacy in real-time clinical workflows. Analyzing individual codes for each AI system may also help pinpoint specific strengths and weaknesses, enabling more targeted improvements. Leveraging metrics designed explicitly for multi-label classification—such as Hamming Loss, Jaccard Index, and Subset Accuracy—would afford a more nuanced view of AI performance. Developing and integrating tailored algorithms that reflect the intricate nature of clinical cases will be crucial for AI systems to manage complex upper-limb fractures effectively. Addressing potential algorithmic biases and ensuring representative training data remain priorities for promoting equitable AI performance, as emphasized by Haider et al. [21]. Assessing the consistency and reliability of different AI systems using metrics like Fleiss’ Kappa or pairwise Cohen’s Kappa can further clarify inter-system agreement.

## 5. Conclusions

This study highlights the varying effectiveness of AI-driven decision support tools in managing hand fractures. ChatGPT emerges as a highly accurate system with strong recall, offering comprehensive coverage of correct interventions at the expense of occasionally proposing superfluous codes. DeepSeek strikes a balance between precision and recall but may overlook some interventions, while Gemini’s significantly lower performance underscores the urgent need for substantial algorithmic and data-driven improvements. These findings contribute to the evolving understanding of AI’s potential in clinical decision-making, demonstrating that while AI systems can enhance care, their integration into routine clinical workflows must be approached with a careful assessment of their strengths and limitations. By addressing algorithmic biases, refining precision without undermining recall, and conducting rigorous validation studies, AI in healthcare can progress toward fulfilling its promise of improved patient outcomes, more efficient workflows, and an elevated standard of personalized medicine in the domain of hand trauma and beyond.

## Figures and Tables

**Table 1:** Patients’ characteristics and their management advice by surgeons and large language models.

Case	Key Details	Proposed ChatGPT Approach	Proposed DeepSeek Approach	Proposed Gemini Approach	Surgeon Approach
1	48 M, punched TV, right 5th MC + little finger mallet	Closed or open reduction with K-wire if unstable, DIP extension for mallet injury	ORIF (Open Reduction Internal Fixation) with volar plating for 5th metacarpal fracture, percutaneous K-wire or screw fixation for bony mallet finger	Splint in slight flexion, closed reduction if significantly displaced, buddy taping for mallet finger	K-wire fixation of 5th MC and mallet finger
2	54 M, Punched dog, right MF and RF mallet fingers	Closed reduction if small bony fragment, K-wire if unstable + DIP splint	Percutaneous K-wire or screw fixation for displaced bony mallet fractures (MF/RF), splinting if non-displaced	Buddy taping for both mallet fingers	K-wire fixation for both mallet fingers
3	14 M, basketball, right MF bony mallet	Closed reduction if the fragment is small, K-wire if unstable, DIP in extension	Extension splinting (6–8 weeks) for non-displaced pediatric bony mallet, percutaneous pinning if displaced	Splint in slight flexion, consider surgical repair if significantly displaced	K-wire fixation of bony mallet fragment + DIP extension splint
4	21 M, punched wall, right 4th MC fracture	Assess displacement, if unstable or significantly angulated, ORIF with plate/screws	CRPP (Closed Reduction Percutaneous Pinning) or ORIF with plates/screws for unstable 4th metacarpal fracture	Splint in slight flexion, closed reduction if significantly displaced	Open reduction and internal fixation (plate and screws) for 4th MC
5	10 M, footy injury, RLF P1 head fracture	Closed reduction or mini-open approach if needed, K-wire fixation if instability, immobilize in extension	CRPP for displaced P1 head fracture, buddy taping for stable, non-displaced fractures; nailbed repair under magnification + K-wire fixation for P3 fracture	Splint in slight flexion, closed reduction if significantly displaced	GAMP technique + K-wire in P1 head
6	69 M, boat vs. LIF crush, LIF P3 fracture + nailbed	Debridement and nailbed repair, K-wire for distal phalanx if unstable	Nailbed repair under magnification + K-wire fixation for P3 fracture	Splint in extension, nailbed repair if necessary	Nailbed repair + K-wire fixation of P3
7	45 M, cricket ball, left RF P2 pilon + P3 mallet	Consider dynamic external fixation (Suzuki) for pilon fracture, K-wire for mallet	ORIF for P2 pilon fracture (mini-fragment screws), splinting or pinning for P3 mallet	Splint in extension, consider surgical repair for pilon fracture	Suzuki + Ishiguru K-wire fixation for pilon (P2) and mallet (P3)
8	7 M, car door, left MF P2/P3 fracture	Closed or open reduction if needed, K-wire stabilization, possible GAMP technique	CRPP for displaced pediatric P2/P3 fractures	Splint in slight flexion, closed reduction if significantly displaced	GAMP + K-wire fixation of P2/P3 MF fractures
9	53 F, dog bite, right IF P1 fracture (open)	Irrigation/debridement due to bite, ORIF if unstable, antibiotics coverage	Urgent irrigation/debridement + ORIF (plates/screws) for open P1 fracture	Irrigation and debridement, antibiotics, tetanus prophylaxis, splint in slight flexion, potential for surgical exploration	Debridement + ORIF of P1, likely with antibiotics
10	49 M, wardrobe door, left 5th MC neck (open)	Washout closed/open reduction, K-wire if angulated/unstable	Debridement + ORIF with plates/screws for open 5th metacarpal neck fracture	Irrigation and debridement, antibiotics, tetanus prophylaxis, splint in slight flexion, potential for surgical exploration	Washout + K-wire fixation (two cross K-wires) of open 5th MC neck
11	9 M, basketball, left LF P1 fracture	Closed or open reduction if displaced, GAMP or K-wire fixation, splinting	CRPP for displaced pediatric P1 fracture	Splint in slight flexion, closed reduction if significantly displaced	GAMP procedure for P1 fracture, likely with protected mobilization
12	69 M, fall from electric scooter, left 3rd and 4th MC fractures	If significantly displaced, ORIF with plates/screws, plus post-op rehab	ORIF with plates/screws for multiple metacarpal fractures	Splint in slight flexion, closed reduction if significantly displaced, consider surgical fixation if multiple fractures	Open reduction and plating of 3rd and 4th metacarpals
13	32 F, hit fridge, right RF P1 fracture	Open reduction if unstable, miniplate or screws fixation, short splint post-op	ORIF (mini-fragment screws) for unstable P1 fracture	Splint in slight flexion, closed reduction if significantly displaced	ORIF (plate and screws or small fragment system) on right RF P1
14	32 M, punched steel beam, right RF and LF MC fractures	Closed or open reduction depending on displacement, GAMP or K-wire if necessary	ORIF for displaced RF/LF metacarpal fractures	Splint in slight flexion, closed reduction if significantly displaced, consider surgical fixation if multiple fractures	GAMP + K-wire fixation of 4th and 5th MC fractures
15	16 M, punch, right 5th MC fracture	Assess displacement, if closed reduction fails, ORIF with plate and screws	CRPP or ORIF (based on angulation >30°)	Splint in slight flexion, closed reduction if significantly displaced	ORIF (plate and screws) on 5th MC + standard rehab protocol
16	35 M, AFL injury, left RF P1 fracture	Open reduction if significantly displaced, stable fixation with miniplate or screws	ORIF with mini-fragment screws for unstable P1 fracture	Splint in slight flexion, closed reduction if significantly displaced	ORIF (plate and screws) on left RF P1 + guided physiotherapy
17	47 F, punch, left 5th MC fracture	Check the degree of angulation, if unstable then ORIF, follow-up X-rays	ORIF with volar plating for 5th metacarpal fracture	Splint in slight flexion, closed reduction if significantly displaced	ORIF (plate and screws) on 5th MC + standard post-op rehab
18	32 M, Futsal ball, right 1st MC fracture	If unstable base/shaft fracture, open reduction and fix with plate or K-wire, then splint	ORIF (Bennett’s fracture protocol: lag screws or K-wires)	Splint in slight flexion, closed reduction if significantly displaced	ORIF + additional K-wire for the stability of the 1st MC
19	6 M, football, DOS 25/4, left RF P1 neck	Closed/mini-open reduction, K-wire if unstable, protect growth plate	CRPP for pediatric P1 neck fracture	Splint in slight flexion, closed reduction if significantly displaced	Left RF P1 neck GAMP
20	22 M, punch, DOS 28/4, right IF MC fracture	Closed or open reduction if unstable, GAMP + K-wire, short immobilization	ORIF with plates/screws for unstable metacarpal fracture	Splint in slight flexion, closed reduction if significantly displaced	Right IF MC GAMP + K-wire
21	19 F, AFL fist vs. ground, DOS 1/5, Right RF MC neck	Closed/open reduction for displacement, GAMP + K-wire for MC neck	CRPP or ORIF for metacarpal neck fracture (based on rotation/angulation)	Splint in slight flexion, closed reduction if significantly displaced	Right RF MC neck GAMP + K-wire
22	14 F, AFL, DOS 1/5, right LF P1 radial condyle	Open reduction to restore joint, small screws/plate fixation	ORIF with mini-screws for radial condyle fracture	Splint in appropriate position, consider further imaging (MRI) if neurovascular compromise	ORIF radial condyle of LF P1
23	15 M, football, DOS 1/5, right LF P1 fracture	Check displacement, GAMP for P1, protect growth plate, short immobilization	CRPP for pediatric P1 fracture	Splint in slight flexion, closed reduction if significantly displaced	Right LF P1 GAMP
24	76 M, FOOSH, DOS 3/5, left thumb P3 fracture	Open/closed reduction if unstable, K-wire, splint afterwards	Splinting for non-displaced P3, K-wire fixation if displaced	Splint in extension, consider surgical repair if significantly displaced	Left thumb P3 open reduction + K-wire
25	9 F, FOOSH, DOS 7/5, left LF MC fracture	Check growth plate, if displaced open reduction + K-wire, short cast	CRPP for pediatric metacarpal fracture	Splint in slight flexion, closed reduction if significantly displaced	Left LF MC open reduction + K-wire
26	22 F, FOOSH, DOS 7/5, left thumb MC base	If the base is unstable (e.g., Bennett-type), open reduction + plate/screws	ORIF (e.g., lag screws for Rolando/Bennett’s fractures)	Splint in appropriate position, consider surgical fixation if unstable	Left thumb MC base ORIF
27	17 M, basketball, DOS 25/4, right thumb Bennett’s	Open/closed reduction for Bennett’s fracture, GAMP or plate, K-wire if unstable	ORIF with screws/K-wires for Bennett’s fracture	Closed reduction and percutaneous pinning	Right thumb Bennett’s GAMP + K-wire
28	9 M, AFL, DOS 11/5, right ring finger P2 neck	If significantly angulated, GAMP fixation, protect growth plate	CRPP for pediatric P2 neck fracture	Splint in slight flexion, closed reduction if significantly displaced	Right ring finger P2 neck GAMP
29	33 F, FOOSH, DOS 11/5, left RF fracture	Open reduction if unstable, plate/screws or K-wire, short splint	ORIF for unstable RF fracture	Splint in slight flexion, closed reduction if significantly displaced	Left RF ORIF (plate/screws)
30	59 M, AFL, DOS 11/5, right LF central slip + volar P2	Repair central slip, fix volar P2 if needed (K-wire), open approach	Central slip tendon repair + ORIF for volar P2 fracture	Splint in extension, consider surgical repair	Right LF central slip repair + K-wire volar P2
31	23 M, punched fridge, DOS 15/5, left 5th MC neck	Check angulation, GAMP or K-wire if unstable, short immobilization	ORIF with plates/screws for 5th metacarpal neck fracture	Splint in slight flexion, closed reduction if significantly displaced	Left 5th MC neck GAMP
32	33 M, crush steel, DOS 18/5, right IF P2 fracture	Washout if open, K-wire if unstable fragment, consider antibiotics	ORIF for unstable P2 fracture	Splint in slight flexion, closed reduction if significantly displaced	Right IF P2 washout + K-wire
33	17 F, AFL, DOS 18/5, left IF MCPJ dislocation	Open reduction of MCPJ, volar plate repair (Kaplan’s lesion), possible pin	Closed reduction ± K-wire fixation for MCPJ dislocation	Closed reduction under anesthesia, splint in appropriate position	Left IF MCPJ dislocation reduction + open volar plate repair
34	26 M, AFL, DOS 22/5, left IF P1 fracture	If displaced or articular, open reduction + screws/plate, short immobilization	ORIF for displaced P1 fracture	Splint in slight flexion, closed reduction if significantly displaced	Left IF P1 ORIF
35	59 M, circular saw, DOS 25/5, left MF nailbed + P3	Debridement, nailbed repair, K-wire distal phalanx if unstable, antibiotics	Nailbed repair + P3 K-wire fixation	Nailbed repair, splint in extension	Left MF nailbed repair + K-wire P3
36	25 M, AFL, DOS 29/5, left LF P1	If the fracture is unstable, ORIF with miniplate/screws, short immobilization	ORIF with mini-fragment screws for P1 fracture	Splint in slight flexion, closed reduction if significantly displaced	Left LF P1 ORIF
37	26 F, dodgeball, DOS 29/5, right LF P1	Open reduction if needed, stable fixation (plate/screws), short splint	ORIF for displaced P1 fracture	Splint in slight flexion, closed reduction if significantly displaced	Right LF P1 ORIF with rigid fixation
38	11 M, basketball, DOS 30/5, left LF P2 SH II	Protect growth plate, closed/mini-open reduction, GAMP or K-wire	CRPP for Salter–Harris II fracture	Splint in extension, consider surgical repair	Left LF P2 SH II GAMP
39	14 M, AFL, DOS 31/5, right MF mallet	Unstable mallet or large fragment → K-wire, else DIP splint	Extension splinting ± percutaneous pinning for bony mallet	Buddy taping to adjacent finger	Right MF mallet K-wire fixation
40	36 F, crush injury, DOS 3/6, left LF P3	Closed/mini-open reduction if needed, K-wire for unstable distal fragment	K-wire fixation or splinting for P3 fracture	Splint in extension, consider surgical repair if significantly displaced	Left LF P3 K-wire fixation
41	19 M, AFL, DOS 4/6, left 5th MC fracture	Assess displacement, if significant, ORIF w/plate/screws, short immobilization	ORIF for 5th metacarpal fracture	Splint in slight flexion, closed reduction if significantly displaced	Left 5th MC ORIF (plate and screws)
42	6 M, AFL, DOS 5/6, left RF fracture	Open/closed reduction for phalanx, K-wire if unstable, protect growth plate	CRPP for pediatric RF fracture	Splint in slight flexion, closed reduction if significantly displaced	Open reduction + K-wires on left ring finger
43	30 F, fall, DOS 5/6, left 5th MC	If the metacarpal neck/shaft is displaced, GAMP or K-wire/plate, short immobilization	ORIF for unstable 5th metacarpal fracture	Splint in slight flexion, closed reduction if significantly displaced	GAMP fixation of left 5th metacarpal
44	63 F, fall, DOS 17/6, left thumb P2	Open reduction if articular, fix w/mini-screws/plate, short splint	ORIF for thumb P2 fracture	Splint in appropriate position, consider surgical repair if significantly displaced	Left thumb P2 ORIF w/hardware
45	39F, 8/6, dog lead, DOS 17/6, right RF P1	Open reduction for displaced P1, small plate or screws, buddy taping	ORIF for unstable P1 fracture	Splint in slight flexion, closed reduction if significantly displaced	Right RF P1 ORIF (plate/screws)
46	30 F, AFL (kicked), DOS 18/6, right thumb P1	Open reduction of proximal phalanx, plate/screws, short thumb splint	ORIF for thumb P1 fracture	Splint in appropriate position, consider surgical repair if significantly displaced	Right thumb P1 ORIF
47	61 F, fall from horse, DOS 21/6, left hamate + capitate	Carpal fractures may need open reduction or GAMP, K-wire or mini-screws ok	ORIF for carpal fractures (hamate + capitate)	Splint in slight flexion, consider surgical fixation	Left hamate + capitate GAMP + K-wire
48	36 M, crush injury, DOS 21/6, right 5th MC	Check displacement, if unstable, GAMP or K-wire, short immobilization	ORIF for 5th metacarpal fracture	Splint in slight flexion, closed reduction if significantly displaced	Right 5th MC GAMP + K-wire
49	28 M, basketball, DOS 23/6, left LF P3 base	If large or joint-involved fragment, GAMP or K-wire, DIP splint	K-wire fixation for P3 base fracture	Splint in extension, consider surgical repair if significantly displaced	Left LF P3 base GAMP + K-wire
50	59 F, softball fall, DOS 24/6, left MF open mallet	Open mallet: nailbed repair if needed, K-wire DIP, watch for infection	Debridement + K-wire fixation for open mallet injury	Splint in extension, surgical repair of mallet finger	Left MF NBR + K-wire for open mallet
51	28 M, AFL, DOS 3/7, left LF PIPJ fracture/dislocation	Open reduction to restore joint, K-wire if unstable, volar plate repair	ORIF ± external fixation for PIPJ fracture-dislocation	Closed reduction under anesthesia, splint in appropriate position	Left LF PIPJ open reduction + K-wire + VP repair
52	72 M, cricket ball, DOS 3/7, right MF P3 fracture	Mallet-type or distal phalanx fracture → K-wire if unstable, DIP splint otherwise	Splinting for non-displaced P3 fracture	Splint in extension, consider surgical repair if significantly displaced	Right MF P3 K-wire fixation
53	23 M, basketball, DOS 10/7, right 5th MC	If significantly unstable, open reduction w/plate/screws or GAMP, short splint	ORIF for 5th metacarpal fracture	Splint in slight flexion, closed reduction if significantly displaced	Right 5th MC ORIF w/hardware
54	28 M, axial load, DOS 12/7, left IF P1 fracture	Open reduction if displaced/intra-articular, mini-plate or screws, early mobilization	ORIF for unstable P1 fracture	Splint in slight flexion, closed reduction if significantly displaced	Left IF P1 ORIF (plate/screws)
55	14 M, basketball, DOS 28/7, right RF distal injury	Nailbed repair or debridement if open, K-wire for unstable distal phalanx, splint DIP	CRPP for distal phalangeal injury	X-ray to assess extent of injury, splint or cast depending on findings	Right RF NBR + P3 K-wire
56	64 M, cricket ball, DOS 25/7, left MF PIPJ dislocation	Re-open if residual subluxation, volar plate repair, possible K-wire across PIPJ	Closed reduction ± K-wire fixation for PIPJ dislocation	Closed reduction under anesthesia, splint in extension	Re-open reduction of left MF PIPJ + VP repair
57	15 M, basketball, DOS 24/7, right MF P2 fracture	Open reduction if displaced/intra-articular, small plate/screws, short immobilization	ORIF for displaced P2 fracture	Splint in slight flexion, closed reduction if significantly displaced	Right MF P2 ORIF
58	30 M, punched TV, DOS 19/7, right 4th and 5th MC + hamate	Check 4th/5th MC alignment + hamate, K-wire or GAMP if unstable, short cast	ORIF for 4th/5th metacarpal and hamate fractures	Splint in slight flexion, consider surgical fixation for multiple fractures	Right 4th + 5th GAMP + hamate K-wire

Abbreviation: MC = Metacarpal; MF = Middle Finger; LF = Left Finger; RF = Right Finger; P1, P2, P3 = Proximal, Middle, and Distal Phalanges; DIP = Distal Interphalangeal; IF = Interphalangeal; ORIF = Open Reduction Internal Fixation; CRPP = Closed Reduction Percutaneous Pinning; GAMP = specific surgical technique for fracture stabilization; K-wire = Kirschner Wire; SH II = Salter–Harris Type II; MCPJ = Metacarpophalangeal Joint; VP repair = Volar Plate Repair; NBR = Nail Bed Repair; DOS = Date of Surgery; AFL = Australian Football League.

**Table 2:** Coded interventions.

Case	Proposed Code by ChatGPT	Proposed Code by DeepSeek	Proposed Code by Gemini	Surgeon Approach
1	1 + 2 + 3 + 5	2 + 4 + 3	6 + 1 + 7	3
2	1 + 3 + 5	3 + 5	7	3
3	1 + 3 + 5	5 + 3	6 + 2	3 + 5
4	2 + 4	9 + 2 + 4	6 + 1	2 + 4
5	1 + 2 + 3 + 5	9 + 7	10 + 3	8 + 3
6	11 + 10 + 3	10 + 3	5 + 10	10 + 3
7	17 + 3	2 + 4 + 5 + 3	5 + 2 + 4	17 + 3
8	1 + 2 + 3 + 8	9	6 + 1	8 + 3
9	11 + 2 + 4 + 12	11 + 2 + 4	11 + 12 + 13 + 6 + 2	11 + 2 + 4 + 12
10	11 + 1 + 2 + 3	11 + 2 + 4	11 + 12 + 13 + 6 + 2	11 + 3
11	1 + 2 + 8 + 3	9	6 + 1	8
12	2 + 4 + 16	2 + 4	6 + 1 + 2 + 4	2 + 4
13	2 + 4 + 6	2 + 4	6 + 1	2 + 4
14	1 + 2 + 8 + 3	2 + 4	6 + 1	8 + 3
15	1 + 2 + 4	9 + 2 + 4	6 + 1	2 + 4 + 16
16	2 + 4	2 + 4	6 + 1	2 + 4 + 16
17	2 + 4	2 + 4	6 + 1	2 + 4 + 16
18	2 + 4 + 3	2 + 4	6 + 1	2 + 4 + 3
19	1 + 2 + 3	9	6 + 1	8
20	1 + 2 + 8 + 3	2 + 4	6 + 1	8 + 3
21	1 + 2 + 8 + 3	9 + 2 + 4	6 + 1	8 + 3
22	2 + 4	2 + 4	6 + 1	2 + 4
23	1 + 8	9	6 + 1	8
24	1 + 2 + 3 + 5	3 + 5	5 + 2	2 + 3
25	2 + 3	9	6 + 1	2 + 3
26	2 + 4	2 + 4	2 + 4	2 + 4
27	1 + 2 + 4 + 8 + 3	2 + 4 + 3	1 + 3	8 + 3
28	1 + 8	9	6 + 1	8
29	2 + 4 + 3	2 + 4	6 + 1	2 + 4
30	14 + 3 + 2	14 + 2 + 4	5 + 2	14 + 3
31	1 + 8 + 3	2 + 4	6 + 1	8
32	11 + 3 + 12	2 + 4	6 + 1	11 + 3
33	2 + 15	1 + 3	1 + 5	2 + 15
34	2 + 4	2 + 4	6 + 1	2 + 4
35	11 + 10 + 3 + 12	10 + 3	10 + 5	10 + 3
36	2 + 4	2 + 4	6 + 1	2 + 4
37	2 + 4	2 + 4	6 + 1	2 + 4
38	1 + 2 + 8 + 3	9	5 + 2	8
39	3 + 5	5 + 3	7	3
40	1 + 2 + 3	3 + 5	5 + 2	3
41	2 + 4	2 + 4	6 + 1	2 + 4
42	1 + 2 + 3	9	6 + 1	2 + 3
43	8 + 3 + 4	2 + 4	6 + 1	8
44	2 + 4	2 + 4	5 + 2 + 4	2 + 4
45	2 + 4 + 7	2 + 4	6 + 1	2 + 4
46	2 + 4	2 + 4	6 + 1	2 + 4
47	2 + 8 + 3 + 4	2 + 4	6 + 2 + 4	8 + 3
48	1 + 2 + 8 + 3	2 + 4	6 + 1	8 + 3
49	8 + 3 + 5	3	5 + 2	8 + 3
50	10 + 3	11 + 3	5 + 2 + 3	10 + 3
51	2 + 3 + 15	2 + 4 + 17	1 + 5	2 + 3 + 15
52	3 + 5	5	5 + 2	3
53	2 + 4 + 8 + 5	2 + 4	6 + 1	2 + 4
54	2 + 4 + 16	2 + 4	6 + 1	2 + 4
55	10 + 11 + 3 + 5	9	5?	10 + 3
56	2 + 15 + 3	1 + 3	1 + 5	2 + 15
57	2 + 4	2 + 4	6 + 1	2 + 4
58	3 + 8	2 + 4	6 + 1 + 2 + 4	8 + 3

This table includes four columns. The “Case” column is a sequential identifier from 1 to 58. The “Proposed Code” is a numeric code representing the treatment approach recommended by the AI or protocol: 1 corresponds to “Closed reduction,” 2 to “Open reduction,” 3 to “K-wire fixation,” 4 to “ORIF (plate/screws),” 5 to “Splint in extension,” 6 to “Splint in flexion,” 7 to “Buddy taping,” 8 to “GAMP technique,” 9 to “CRPP (Closed Reduction Percutaneous Pinning),” 10 to “Nailbed repair,” 11 to “Debridement/Irrigation (washout),” 12 to “Antibiotic coverage,” 13 to “Tetanus prophylaxis,” 14 to “Central slip tendon repair,” 15 to “Volar plate repair,” 16 to “Physiotherapy/Rehabilitation,” and 17 to “External fixation (e.g., Suzuki frame). The “Surgeon approach” column uses the same coding scheme to indicate the procedure that was actually performed.

**Table 3:** Statistical analysis.

AI	Accuracy (%)	Precision (%)	Recall (%)	F1 Score (%)
ChatGPT	98.28	58.48	92.14	71.38
DeepSeek	64.19	61.17	58.29	59.52
Gemini	19.37	13.00	15.08	13.55

## Data Availability

The data are available upon reasonable request to the corresponding author.

## Abbreviations

The following abbreviations are used in this manuscript:

AI	Artificial Intelligence
LLM	Large Language Model
MC	Metacarpal
MF	Middle Finger
LF	Little Finger
RF	Ring Finger
P1, P2, P3	Proximal, Middle, and Distal Phalanges
DIP	Distal Interphalangeal Joint
IF	Index Finger
ORIF	Open Reduction and Internal Fixation
CRPP	Closed Reduction Percutaneous Pinning
GAMP	Guided Anatomic Mini-invasive Procedure
K-wire	Kirschner Wire
SH II	Salter–Harris Type II
MCPJ	Metacarpophalangeal Joint
VP Repair	Volar Plate Repair
NBR	Nail Bed Repair
DOS	Date of Surgery
AFL	Australian Football League
